# Triglyceride glucose index was linearly associated with abdominal aortic calcification based on NHANES 2013–2014

**DOI:** 10.1186/s12902-022-01226-w

**Published:** 2022-12-15

**Authors:** Ying Zhou, Fu Zhi, Beibei Gao, Shengen Liao

**Affiliations:** Department of Cardiology, The First Affiliated Hospital of Nanjing Medical University, Nanjing, 210029 Jiangsu China

**Keywords:** Triglyceride glucose index, Abdominal aortic calcification, Insulin resistance

## Abstract

**Purpose:**

To study the relationship between the TyG index and the risk of AAC.

**Methods:**

We enrolled 1,486 participants from the National Health and Nutrition Examination Survey (NHANES). The TyG index was calculated in the log-transformed of triglycerides multipled by glucose, and the presence of AAC was diagnosed as AAC score above than 0.

**Results:**

Our suggested found that TyG level was positively correlated with the presence of AAC and log-transformed AAC score. After adjusted for other variables, comparing with the lowest quartile of TyG index, the highest quartile of TyG level was significantly associated with the presence of AAC (OR 2.12, 95%CI 1.05–4.35, *p* = 0.038) and severe AAC (OR 2.12, 95%CI 1.05–4.35, *p* = 0.038).

**Conclusions:**

TyG index was significantly associated with the risk of AAC and severe AAC, which could be a marker in clinical practice.

**Supplementary Information:**

The online version contains supplementary material available at 10.1186/s12902-022-01226-w.

## Introduction

Cardiovascular disease (CVD) is a leading cause of death worldwide [1]. Abdominal aortic calcification (AAC), as measured by dual-energy X-ray absorptiometry (DXA) [2], has been reported to be an independent marker for atherosclerotic vascular diseases [3] and a predictor for all-cause mortality and cardiovascular events [4, 5]. The Framingham study indicated that AAC was present in 15.5% of men and 7.8% in women under 45 years age, however, the prevalence increased to 100% in both men and women over 75 years [6]. On one hand, calcification of arteries is increasingly seen as a protective factor, preventing plaque rupture and aneurysm growth of abdominal aorta. On the other hand, it is also an end stage of a dangerous inflammatory process. Exploring the factor associated with the presence of AAC is of great importance to identify the risk factors of AAC for reducing the complications.

The triglyceride glucose (TyG) index has been proposed as a reliable surrogate marker of IR [7, 8]. This index is correlated with IR and homeostatic model assessment of insulin resistance (HOMA-IR) [9, 10]. It was reported that TyG index was associated with increased the risk of arterial stiffness and coronary arthery calcification [11, 12]. Many studies also have identified TyG index was a predictor of CVD [13, 14]. However, no study has invesitgated the relationship between the TyG index and AAC in healthy adults.

Therefore, in the present study, we aimed to evaluate the association between the TyG index and the risk of AAC in healthy US adults, which could be a common and accessible marker in clinical practice.

## Methods

### Study population

The National Health and Nutrition Examination Survey (NHANES) is a nationally representative survey performed by the Centers for Disease Control and Prevention. All participants provided written informed consent. A total of 10,157 subjects were enrolled in the NHANES 2013–2014. After excluding those without AAC score records and with missing data on glucose or triglyceride, 1486 participants were included in the current study. We have compared the difference between responders and non-responders and the difference of baseline variabels were comparebale. The study protocol was approved by the Ethics Review Board of National Center for Health Statistics and all participants provided written informed consent.

### Covariate definition and AAC

Information on age, sex, race/ethnicity, education level, family income-povery ratio, smoking status, physical activity, hypertension or diabetes were collected by using standardized questionnaires. Blood glucose, triglyceride, calcium, and phosphorus were measured by standard biochemistry assay. The TyG index was determined as ln (triglycerides [mg/dL] × glucose [mg/dL]/2). The AAC score were calculated using a Kauppila score system according to lateral lumbar spine dual-energy X-ray absorptiometry (DXA) (https://wwwn.cdc.gov/Nchs/Nhanes/2013–2014/DXXAAC_H.htm). The presence of AAC was diagnosed as AAC above than 0 and severe AAC above than 6. All methods were performed in accordance with the relevant the Declaration of Helsinki.

### Statistical analysis

Multivariable logistic regressions were used to examine the association between TyG index, as a continuous or categorical variable, and the risk of AAC. Subgroup analyses were performed to investigate the interactive variables mediating the association between TyG and AAC, including age, gender, BMI, presence of diabetes or hypertension. To explore the nonlinear relationship between TyG index and AAC, we performed the restricted cubic spline curves with 3 knots (0.10, 0.50 and 0.90 respectively). Besides, we added some sensitivity analysis. To explore the association between TyG index with severe AAC, we defined the presence of severe AAC as Kauppilia score > 6 [15]. To explore the linear relationship between the TyG index and Kauppilia score, we performed multivariable liner regression analysis to examinze the relationship between TyG index and log-transformed AAC score [16]. Due to a higher prevalence of AAC in the population (> 20%), we performed Cox proportional hazard regression models with adding a constant time variable [17]. Data were analyzed using R sofeware version 3.6.0. *P* value < 0.05 was considered as statistically significant.

## Results

Participants were stratified into four groups according to their TyG quantiles. The baseline clinical and laboratory characteristics were shown in the enrolled population (Table 1) and excluded population (Supplementary Table 1). The highest TyG quantile tended to have more percentage of male, non-Hispanic white, obesity and diabetes. In addition, the prevalence of AAC significantly increased with an increase in the TyG index.

**Table 1:** Baseline characteristics of the study population according to TyG index in NHANES 2013–2014. Date were expressed as number ± standard deviation for continuous variables or number (percentage) for categorical variables

	Q1(lowest)	Q2	Q3	Q4(highest)	*P* value
N	372	371	371	372	
Age (years)	57.7 ± 12.9	59.5 ± 12.3	59.4 ± 11.7	59.0 ± 11.7	< 0.001
Male (%)	149 (40.1)	183 (49.3)	183 (49.3)	207 (55.6)	0.031
Race/ethnicity (%)					< 0.001
Non-Hispanic white	160 (43.0)	177 (47.7)	148 (39.9)	187 (50.3)	
Non-Hispanic black	110 (29.6)	76 (20.5)	52 (14.0)	38 (10.2)	
Mexican American	32 (8.6)	35 (9.4)	64 (17.3)	38 (10.2)	
Other	70 (18.8)	83 (22.4)	107 (28.8)	87 (23.4)	
Education levels (%)					0.090
Less than high school	74 (19.9)	82 (22.1)	108 (29.1)	97 (26.1)	
High school or equivalent	82 (22.1)	77 (20.8)	75 (20.2)	82 (22.1)	
College or above	215 (58.0)	212 (57.1)	188 (50.7)	192 (51.8)	
Family income- poverty ratio (%)					0.003
≤ 1.0	64 (18.7)	56 (15.9)	79 (23.3)	64 (18.9)	
1.0–3.0	125 (36.4)	116 (32.9)	135 (39.8)	144 (42.5)	
> 3.0	154 (44.9)	181 (51.3)	25 (36.9)	131 (38.6)	
BMI (kg/m^2^)					< 0.001
< 25.0	163 (44.1)	126 (34.3)	85 (23.1)	45 (12.1)	
25.0–29.9	124 (33.5)	140 (38.1)	152 (41.3)	145 (39.1)	
≥ 30.0	83 (22.4)	101 (27.5)	131 (35.6)	181 (48.8)	
Smoking status (%)					0.213
Current smoker	50 (18.1)	60 (21.7)	65 (25.3)	66 (25.4)	
Ever smoker	6 (2.2)	11 (4.0)	9 (3.5)	11 (4.2)	
Never smoker	221 (79.8)	206 (74.4)	183 (71.2)	183 (70.4)	
Physical activity (%)					0.695
Vigorous	61 (32.6)	55 (29.7)	55 (31.2)	71 (37.0)	
Moderate	64 (34.2)	72 (38.9)	68 (38.6)	70 (36.5)	
Inactive	62 (33.2)	58 (31.4)	53 (30.1)	51 (26.6)	
Hypertension (%)	63 (17.7)	75 (20.7)	84 (23.3)	82 (22.5)	0.260
Diabetes (%)	21 (5.7)	41 (11.1)	61 (16.4)	158 (42.5)	< 0.001
Osteoporosis (%)	28 (7.5)	33 (8.9)	41 (11.1)	31 (8.3)	0.375
Glucose(mg/dL)	96.1 ± 11.6	102.1 ± 15.8	108.6 ± 22.5)	138.9 ± 59.2	< 0.001
Triglyceride (mg/dL)	54.5 ± 12.7	86.3 ± 15.2	122.4 ± 22.6	235.2 ± 232.9	< 0.001
TyG index	7.83 ± 0.26	8.36 ± 0.12	8.77 ± 0.13	9.51 ± 0.47	< 0.001
HOMA-IR	0.25 ± 0.19	0.39 ± 0.41	0.53 ± 0.84	1.18 ± 2.70	< 0.001
Calcium (mmol/L)	2.34 ± 0.09	2.35 ± 0.09	2.36 ± 0.08	2.36 ± 0.09	0.004
25-VitD3 (nmol/L)	33.51 ± 37.77	30.09 ± 36.12	34.43 ± 38.94	24.94 ± 34.27	0.003
Phosphorus (mmol/L)	1.21 ± 0.18	1.21 ± 0.16	1.21 ± 0.18	1.20 ± 0.18	0.594
eGFR (ml/min per 1.73m^2^)	92.9 ± 34.0	92.9 ± 33.5	96.6 ± 34.8	104.9 ± 40.1	< 0.001
AAC (%)	88 (23.7)	114 (30.7)	138 (37.2)	137 (36.8)	< 0.001

*BMI* Body mass index, *TyG* Triglyceride glucose index, *HOMA-IR* eGFR estimated glomerular filtration rate, *AAC* Abdominal aortic calcification

**Fig. 1:**
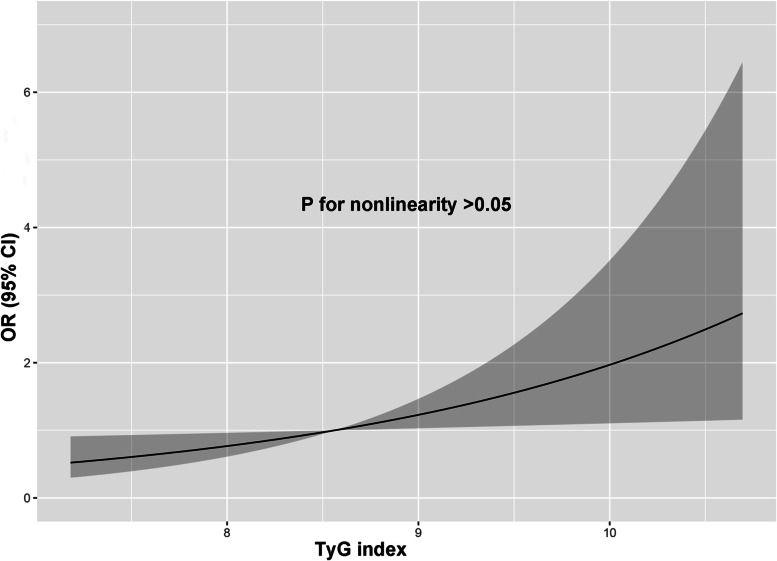
The dose–response relationships between the TyG index and AAC based on a cubic restricted spine curves in the fully-adjusted model. Solid lines are multivariable adjusted hazard ratios, with shadow lines showing 95% confidence intervals derived from restricted cubic spline regressions

To explore whether there is a presence of nonlinearity between TyG index and AAC, we perfomed a restricted cubic anlysis based on logistic regression models. As shown in Fig. 1, in the fully-adjusted models, TyG index was linearly associated with the presence of AAC. Therefore, multivariable logistic regression was used to evaluate the relationship between the TyG index and ACC by categorizing the TyG index into quartiles and using the first quartile as the reference (Table 2). In model 1, the highest TyG quantile was associated with a higher presence of AAC (OR 1.83, 95%CI 1.31–2.58; *p* < 0.001). The OR for having AAC in the highest TyG quartile was 2.08 (95%CI 1.08–4.08; *p* = 0.031). In the fully adjusted model, the association still existed (OR 2.12, 95%CI 1.05–4.35; *p* = 0.038). The coefficients and *P* values for each variable entered into the model 3 were listed in Supplementary Table 2. To explore the association between TyG index with severe AAC, we defined the presence of severe AAC as Kauppilia score > 6. As shown in Table 3, the highest quartile of TyG index was also significantly associated with severe AAC (OR 4.69, 95%CI 1.84–8.35; *p* = 0.009)). Besides, to explore the linear relationship between the TyG index and Kauppilia score, we performed multivariable liner regression analysis to examinze the relationship between TyG index and log-transformed AAC score. As shown in Table 4, TyG index was positively related to Kauppilia score (β = 0.17, 95%CI 0.06–0.27; *p* = 0.002). Due to a higher prevalence of AAC in the population (> 20%), we performed Cox proportional hazard regression models with adding a constant time variable. As shown in Table 5, a higher level of TyG index was associated with a higher risk of AAC.

**Table 2:** Odds ratios and 95% confidence intervals for AAC according to TyG index

	OR (95% CI) *P*			
	Q1	Q2	Q3	Q4
**Model 1**	1.00	1.30 (0.92–1.84) *P* = 0.139	1.83 (1.31–2.57) *P* < 0.001	1.83 (1.31–2.58) *P* < 0.001
**Model 2**	1.00	0.92 (0.48–1.76) *P* = 0.792	2.05 (1.05–4.06) *P* = 0.037	2.08 (1.08–4.08) *P* = 0.031
**Model 3**	1.00	0.92 (0.47–1.80) *P* = 0.816	1.99 (0.99–4.07) *P* = 0.056	2.12 (1.05–4.35) *P* = 0.038

Model 1: adjusted for age, and sex

Model 2: further adjusted for race, edu, IPR, smoking, BMI, and physical activity

Model 3: further adjusted for hypertension, diabetes, calcium, phosphorus, and eGFR

*OR* Odds ratio, *95% CI* 95% confidence interval

**Table 3:** The odds ratios and 95% confidence intervals for severe AAC according to TyG index

	OR (95% CI) *P*			
	Q1	Q2	Q3	Q4
**Model 1**	1.00	1.08 (0.61–1.94) *P* = 0.786	1.84 (1.08–3.21) *P* = 0.028	1.65 (1.05–2.90) *P* = 0.045
**Model 2**	1.00	1.37 (0.27–7.72) *P* = 0.700	5.62 (1.38–9.58) *P* = 0.024	4.19 (2.12–8.12) *P* = 0.005
**Model 3**	1.00	1.30 (0.25–7.41) *P* = 0.752	5.29 (1.22–9.31) *P* = 0.036	4.69 (1.84–8.35) *P* = 0.009)

Model 1: adjusted for age, and sex

Model 2: further adjusted for race, edu, IPR, smoking, BMI, and physical activity

Model 3: further adjusted for hypertension, diabetes, calcium, phosphorus, and eGFR

*OR* Odds ratio, *95% CI* 95% confidence interval

**Table 4:** The coefficient and 95% confidence intervals for log-transformed AAC score according to TyG index

	β	95% confidence interval	P
Model 1	0.09	0.03–0.15	0.002
Model 2	0.15	0.06–0.25	0.002
Model 3	0.17	0.06–0.27	0.002

Model 1: adjusted for age, and sex

Model 2: further adjusted for race, edu, IPR, smoking, BMI, and physical activity

Model 3: further adjusted for hypertension, diabetes, calcium, phosphorus, and eGFR

**Table 5:** The odds ratios and 95% confidence intervals for AAC according to TyG index based on Cox proportional hazard regression modes

	OR (95% CI) *P*			
	Q1	Q2	Q3	Q4
**Model 1**	1.00	1.24 (0.94–1.64) *P* = 0.134	1.59 (1.21–2.07) *P* = 0.001	1.60 (1.23–2.10) *P* = 0.001
**Model 2**	1.00	0.94 (0.55–1.62) *P* = 0.821	1.72 (1.01–2.93) *P* = 0.045	1.80 (1.06–3.06) *P* = 0.029
**Model 3**	1.00	0.96 (0.56–1.67) *P* = 0.892	1.80 (1.05–3.12) *P* = 0.034	1.95 (1.11–3.43) *P* = 0.020)

Model 1: adjusted for age, and sex

Model 2: further adjusted for race, edu, IPR, smoking, BMI, and physical activity

Model 3: further adjusted for hypertension, diabetes, calcium, phosphorus, and eGFR

*OR* Odds ratio, *95% CI* 95% confidence interval

**Fig. 2:**
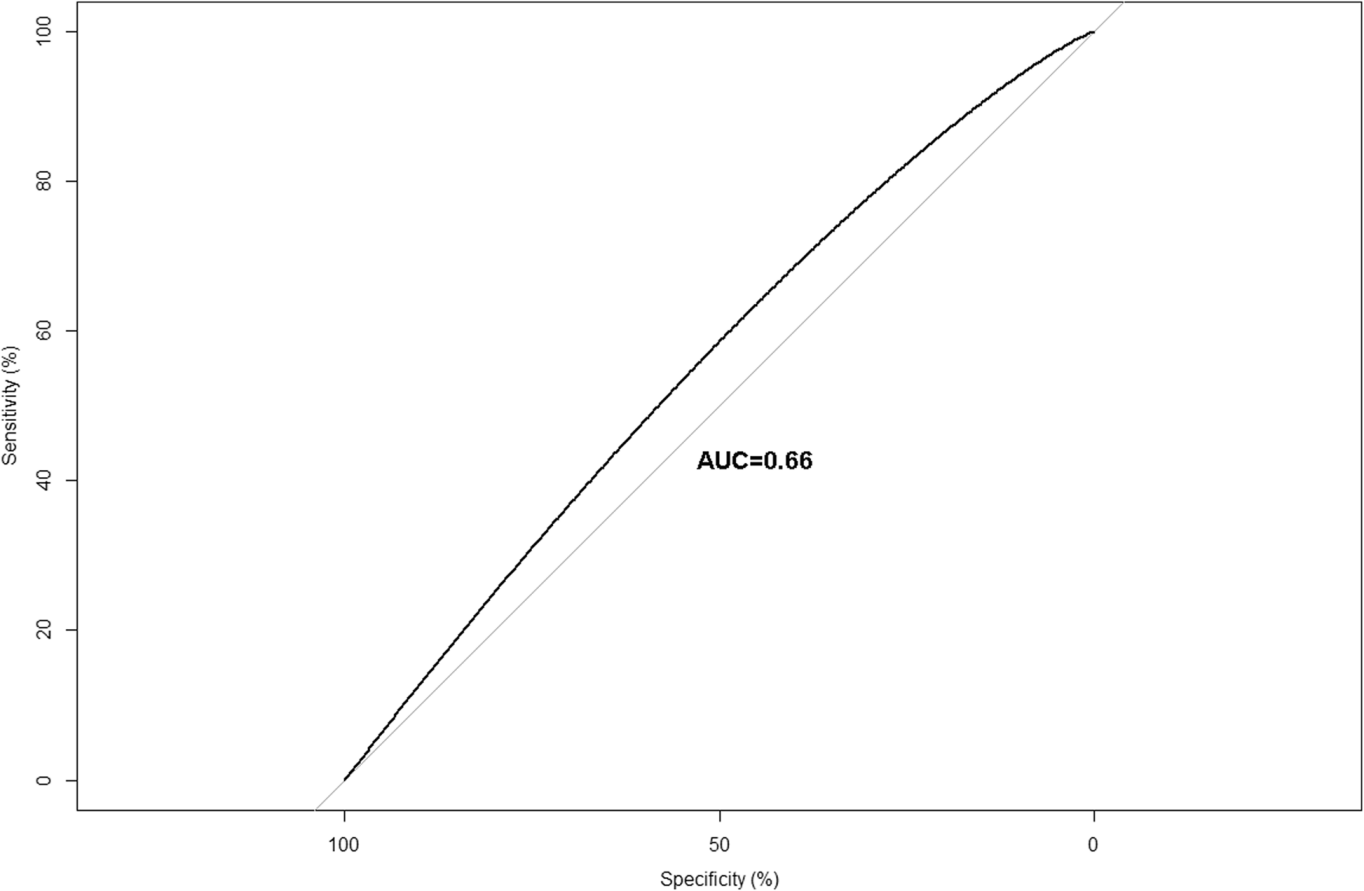
The ROC of the diagnostic capacity of TyG index for the presence of AAC

Subgroup analysis for the associations between TyG index and the presence of AAC was shown in Table 6. The association was consistent across gender, BMI categories and chronic illness. No significant interactions were observed except for age. The association was more stronger in elderly (age > 60 years old). Even though a higher odds ratio in female individuals, the interaction was not significant. Finally, ROC analysis suggested that TyG index could be a predictor of the presence of AAC (AUC = 0.66) (Fig. 2).

**Table 6:** Subgroups analysis for the associations between TyG index and the presence of AAC

	OR (95% CI)	*P* for interaction
**Age**		0.008
≤ 60	1.04 (0.96–1.12)	
> 60	**1.21 (1.03–1.41)**	
**Gender**		0.657
Female	1.06 (0.96–1.18)	
Male	1.10 (1.00–1.21)	
**Hypertension**		0.985
No	1.05 (0.97–1.13)	
Yes	1.13 (0.94–1.35)	
**Diabetes**		0.546
No	1.08 (1.00–1.17)	
Yes	1.08 (0.89–1.32)	
**BMI**		0.979
< 25.0	1.10 (0.96–1.26)	
25.0–29.9	1.14 (1.02–1.27)	
≥ 30.0	1.01 (0.89–1.15)	

Analyses were adjusted in Model 3 except for the strata variables

## Discussion

We found that a higher TyG index was significantly associated with the prevalence of AAC and severe AAC. Besides, the association was consistent across gender, BMI categories and chronic illness. ROC analysis suggested that TyG index could be a predictor of the presence of AAC.

The TyG index has also been proved to be useful for identifying adult population at a high risk of cardiovascular disease [18] and predicting adverse outcomes in patients with type 2 diabetes and CVD [13].In addition, some studies showed that the TyG index was significantly associated with the severity of coronary artery stenosis [19] and artery stiffness [20]. In consistent with previous results that the TyG index associated with coronary artery calcification [21, 22], we confirmed a relationship between the TyG index and abdominal artery calcification.

Because basic anthropometrics have been found to be powerful predictors of mortality compared with cardiometabolic risks [23], when investgating the association between the TyG index, as an easy-to-obtain indicator and artery calcification, we adjusted the cardiovascular risk factors like in most studies [20, 22, 24]. Besdies, we also further adjusted the biochemical parameters including calcium, phosphorus and eGFR, excluding the confounding effect of calcium and phosphorus metabolism. Finally, subgroup analysis found an interactional effect between age and the TyG index. In summary, the mechanism undelying the relationship could be linked to IR. IR could lead to inflammation and atherosclerosis [25], dampening the distensibility and elasticity of abodominal aorta [26]. Vascular calcification was highly prevalent and, when present, was associated with major adverse cardiovascular events [27].

Some limitations existed in our study. Firstly, this is a cross-sectional study. Secondly, adjustment is partial and residual confounders cannot be ruled out. Finally, data on diet are missing.

## Conclusions

In our study, we demonstrated that the TyG index was independently associated with the risk of AAC and severe AAC. The association was consistent across gender, BMI categories and chronic illness.

## Supplementary Information

**Additional file 1: Supplementary Table 1.** The baseline difference between responders and non-responders according to TyG index. **Supplementary Table 2.** The coefficients and P values for each variable entered into the multivariable logistical analysis in Model 3.

## Data Availability

The datasets were available from NHANES 2013–2014 (https://www.cdc.gov/nchs/nhanes/index.htm).

## References

[1] Evans MA, Sano S, Walsh K (2020). Cardiovascular Disease, Aging, and Clonal Hematopoiesis. Annu Rev Pathol.

[2] Kauppila LI, Polak JF, Cupples LA, Hannan MT, Kiel DP, Wilson PW (1997). New indices to classify location, severity and progression of calcific lesions in the abdominal aorta: a 25-year follow-up study. Atherosclerosis.

[3] Bartstra JW, Mali W, Spiering W, de Jong PA. Abdominal aortic calcification: from ancient friend to modern foe. Eur J Prev Cardiol. 2021;28:1386-91. 10.1177/2047487320919895 34647579

[4] Niu Q, Hong Y, Lee CH, Men C, Zhao H, Zuo L (2018). Abdominal aortic calcification can predict all-cause mortality and CV events in dialysis patients: A systematic review and meta-analysis. PLoS ONE.

[5] Ramirez-Velez R, Garcia-Hermoso A, Correa-Rodriguez M, Lobelo F, Gonzalez-Ruiz K, Izquierdo M (2021). Abdominal aortic calcification is associated with decline in handgrip strength in the U.S. adult population >/=40 years of age. Nutr Metab Cardiovasc Dis.

[6] Kiel DP, Kauppila LI, Cupples LA, Hannan MT, O'Donnell CJ, Wilson PW (2001). Bone loss and the progression of abdominal aortic calcification over a 25 year period: the Framingham Heart Study. Calcif Tissue Int.

[7] Guerrero-Romero F, Villalobos-Molina R, Jimenez-Flores JR, Simental-Mendia LE, Mendez-Cruz R, Murguia-Romero M, Rodriguez-Moran M (2016). Fasting Triglycerides and Glucose Index as a Diagnostic Test for Insulin Resistance in Young Adults. Arch Med Res.

[8] Simental-Mendia LE, Rodriguez-Moran M, Guerrero-Romero F (2008). The product of fasting glucose and triglycerides as surrogate for identifying insulin resistance in apparently healthy subjects. Metab Syndr Relat Disord.

[9] Lee SH, Han K, Yang HK, Kim MK, Yoon KH, Kwon HS, Park YM (2015). Identifying subgroups of obesity using the product of triglycerides and glucose: the Korea National Health and Nutrition Examination Survey, 2008–2010. Clin Endocrinol (Oxf).

[10] Du T, Yuan G, Zhang M, Zhou X, Sun X, Yu X (2014). Clinical usefulness of lipid ratios, visceral adiposity indicators, and the triglycerides and glucose index as risk markers of insulin resistance. Cardiovasc Diabetol.

[11] Lambrinoudaki I, Kazani MV, Armeni E, Georgiopoulos G, Tampakis K, Rizos D, Augoulea A, Kaparos G, Alexandrou A, Stamatelopoulos K (2018). The TyG Index as a Marker of Subclinical Atherosclerosis and Arterial Stiffness in Lean and Overweight Postmenopausal Women. Heart Lung Circ.

[12] Park K, Ahn CW, Lee SB, Kang S, Nam JS, Lee BK, Kim JH, Park JS (2019). Elevated TyG Index Predicts Progression of Coronary Artery Calcification. Diabetes Care.

[13] Zhao Q, Zhang TY, Cheng YJ, Ma Y, Xu YK, Yang JQ, Zhou YJ (2020). Impacts of triglyceride-glucose index on prognosis of patients with type 2 diabetes mellitus and non-ST-segment elevation acute coronary syndrome: results from an observational cohort study in China. Cardiovasc Diabetol.

[14] Wang L, Cong HL, Zhang JX, Hu YC, Wei A, Zhang YY, Yang H, Ren LB, Qi W, Li WY (2020). Triglyceride-glucose index predicts adverse cardiovascular events in patients with diabetes and acute coronary syndrome. Cardiovasc Diabetol.

[15] Wu M, Liu Y, Zhong C, Xu B, Kang L (2021). Osteoporosis was associated with severe abdominal aortic calcification based on a cross-sectional study. Arch Osteoporos.

[16] Liu N, Feng Y, Zhan Y, Ma F (2022). Relationship between blood cadmium and abdominal aortic calcification: NHANES 2013–2014. J Trace Elem Med Biol.

[17] Assareh AA, Haybar H, Malekzadeh H, Yazdanpanah L, Bozorgmanesh M (2014). No Relationship between Serum and Salivary beta2- Microglobulin Levels in A Sample of Adult Diabetic Men with Chronic Kidney Disease without Renal Replacement Therapy. Cell J.

[18] Irace C, Carallo C, Scavelli FB, De Franceschi MS, Esposito T, Tripolino C, Gnasso A (2013). Markers of insulin resistance and carotid atherosclerosis. A comparison of the homeostasis model assessment and triglyceride glucose index. Int J Clin Pract.

[19] Thai PV, Tien HA, Van Minh H, Valensi P (2020). Triglyceride glucose index for the detection of asymptomatic coronary artery stenosis in patients with type 2 diabetes. Cardiovasc Diabetol.

[20] Lee SB, Ahn CW, Lee BK, Kang S, Nam JS, You JH, Kim MJ, Kim MK, Park JS (2018). Association between triglyceride glucose index and arterial stiffness in Korean adults. Cardiovasc Diabetol.

[21] Won KB, Park EJ, Han D, Lee JH, Choi SY, Chun EJ, Park SH, Han HW, Sung J, Jung HO (2020). Triglyceride glucose index is an independent predictor for the progression of coronary artery calcification in the absence of heavy coronary artery calcification at baseline. Cardiovasc Diabetol.

[22] Kim MK, Ahn CW, Kang S, Nam JS, Kim KR, Park JS (2017). Relationship between the triglyceride glucose index and coronary artery calcification in Korean adults. Cardiovasc Diabetol.

[23] Krakauer NY, Krakauer JC (2018). Anthropometrics, Metabolic Syndrome, and Mortality Hazard. J Obes.

[24] Liu Y, Wu M, Xu J, Sha D, Xu B, Kang L (2020). Association between Triglyceride and glycose (TyG) index and subclinical myocardial injury. Nutr Metab Cardiovasc Dis.

[25] Kahn AM, Allen JC, Seidel CL, Zhang S (2000). Insulin inhibits migration of vascular smooth muscle cells with inducible nitric oxide synthase. Hypertension.

[26] Simon SP, Fodor D, Muntean L, Poanta L, Cristea P, Rednic S (2014). Bone mineral density, vertebral fractures and body mass index in postmenopausal women with abdominal aortic calcification. Endocr Res.

[27] Xu Z, Suo CJ, Ruan YS, Tan RY, Zhang W, Niu TL (2018). Effect and related mechanisms of RTA-408 on rat vascular smooth muscle cell calcification induced by advanced glycation end products. Zhonghua Xin Xue Guan Bing Za Zhi.

