# Supplementary material for: Triglyceride glucose index was linearly associated with abdominal aortic calcification based on NHANES 2013–2014
Source: BMC Endocr Disord. 2022 Dec 15;22:318. doi: 10.1186/s12902-022-01226-w (PMC9753247; doi:10.1186/s12902-022-01226-w)
Supplement: Supplementary file 1 — Additional file 1: Supplementary Table 1. The baseline difference between responders and non-responders according to TyG index. Supplementary Table 2. The coefficients and P values for each variable entered into the multivariable logistical analysis in Model 3. [file 12902_2022_1226_MOESM1_ESM.docx]

**Supplementary Table 1.** The baseline difference between responders and non-responders according to TyG index.

| Demographics | Responders (n=1486) | Non-responders (n=8689) |
| --- | --- | --- |
| Male (%) | 722 (48.6) | 4281 (49.2) |
| Race/ethnicity (%) |  |  |
| Non-Hispanic white | 672 (45.2) | 3002 (34.5) |
| Non-Hispanic black | 276 (18.6) | 1991 (22.9) |
| Mexican American | 191 (12.9) | 1539 (17.7) |
| Other | 347 (23.4) | 2157 (24.8) |
| Education levels (%) |  |  |
| Less than high school | 361 (24.3) | 885 (20.7) |
| High school or equivalent | 316 (21.3) | 987 (23.1) |
| College or above | 807 (54.4) | 2406 (56.3) |
| Family income- poverty ratio (%) |  |  |
| ≤1.0 | 263 (19.1) | 2472 (18.4) |
| 1.0-3.0 | 520 (37.8) | 3125 (35.9) |
| >3.0 | 591 (43.0) | 2419 (37.8) |
| Smoking status (%) |  |  |
| Current smoker | 241 (16.2) | 751 (22.9) |
| Ever smoker | 37 (2.5) | 203 (2.3) |
| Never smoker | 793 (53.4) | 2739 (51.5) |
| Physical activity (%) |  |  |
| Vigorous | 242 (32.7) | 1004 (42.1) |
| Moderate | 274 (37.0) | 1194 (50.1) |
| Inactive | 224 (30.3) | 930 (39.0) |

**Supplementary Table 2**. The coefficients and P values for each variable entered into the multivariable logistical analysis in Model 3.

| Variables | OR | 95% CI | P value |
| --- | --- | --- | --- |
| TyG quartile |  |  |  |
| Q1 | ref | - | - |
| Q2 | 0.92 | [0.47, 1.80] | 0.816 |
| Q3 | 1.99 | [0.99, 4.07] | 0.056 |
| Q4 | 2.12 | [1.05, 4.35] | 0.038 |
| Age | 1.04 | [1.01, 1.07] | 0.005 |
| Male | 0.88 | [0.53, 1.45] | 0.614 |
| Race |  |  |  |
| Non-Hispanic white | ref | - | - |
| Non-Hispanic black | 0.66 | [0.33, 1.29] | 0.233 |
| Mexican American | 0.82 | [0.38, 1.72] | 0.605 |
| Other | 0.53 | [0.28, 0.98] | 0.046 |
| Education levels |  |  |  |
| Less than high school | ref | - | - |
| High school or equivalent | 0.99 | [0.50, 1.95] | 0.977 |
| College or above | 0.92 | [0.50, 1.71] | 0.798 |
| IPR |  |  |  |
| ≤1.0 | ref | - | - |
| 1.0-3.0 | 0.77 | [0.42, 1.41] | 0.397 |
| >3.0 | 0.55 | [0.28, 1.07] | 0.076 |
| BMI |  |  |  |
| <25.0 | ref | - | - |
| 25.0-29.9 | 1.43 | [0.81, 2.53] | 0.220 |
| ≥30.0 | 0.83 | [0.39, 1.77] | 0.639 |
| Smoking status |  |  |  |
| Current smoker | ref | - | - |
| Ever smoker | 0.82 | [0.27, 2.37] | 0.724 |
| Never smoker | 0.50 | [0.28, 0.88] | 0.016 |
| Physical activity |  |  |  |
| Vigorous | ref | - | - |
| Moderate | 1.00 | [0.56, 1.78] | 0.995 |
| Inactive | 1.37 | [0.77, 2.46] | 0.291 |
| Hypertension | 0.99 | [0.55, 1.75] | 0.970 |
| DM | 1.26 | [0.67, 2.34] | 0.465 |
| Calcium | 0.20 | [0.01, 2.83] | 0.236 |
| Phosphorus | 0.69 | [0.18, 2.63] | 0.586 |
| eGFR | 0.99 | [0.98, 1.00] | 0.014 |
